# Slowly Progressive Secondary Adrenal Insufficiency Due to Pembrolizumab Administration in a Patient With a History of Pituitary Neuroendocrine Tumor

**DOI:** 10.7759/cureus.81495

**Published:** 2025-03-31

**Authors:** Hiroyuki Ueda, Yukari Fujita, Kosuke Mukai, Kazuyuki Miyashita, Junji Kozawa, Hitoshi Nishizawa, Iichiro Shimomura

**Affiliations:** 1 Department of Metabolic Medicine, Osaka University Graduate School of Medicine, Suita, JPN; 2 Department of Diabetes Care Medicine, Osaka University Graduate School of Medicine, Suita, JPN; 3 Department of Metabolism and Atherosclerosis, Osaka University Graduate School of Medicine, Suita, JPN

**Keywords:** hyponatremia, immune checkpoint inhibitor, immune-related adverse events, pituitary neuroendocrine tumor, secondary adrenal insufficiency

## Abstract

A 70-year-old man developed anorexia, general malaise, and hyponatremia with a serum sodium level of 120 mEq/L after the fifth cycle of pembrolizumab administration for bladder cancer. A rapid adrenocorticotropic hormone (ACTH) loading test result was within the normal range (basal and peak cortisol levels of 8.2 μg/dL and 20.0 μg/dL, respectively), and his serum sodium level recovered by salt loading. However, about two weeks later, his anorexia and malaise worsened, and his serum sodium level decreased to 122 mEq/L. A rapid ACTH loading test performed again four weeks after the first test showed a peak cortisol level of 13.4 μg/dL, and a corticotropin-releasing hormone (CRH) loading test showed basal ACTH and cortisol levels of 19 pg/mL and 6.3 μg/dL and peak levels of 34 pg/mL and 8.7 μg/dL, respectively. Therefore, secondary adrenal insufficiency (SAI) was diagnosed. Although he underwent nonfunctioning pituitary neuroendocrine tumor resection at the age of 55 years, the tumor was not significantly different from the postoperative findings. The possible cause of SAI was ACTH deficiency after pembrolizumab administration. Four months after diagnosis, the basal ACTH and cortisol levels had decreased from 19 to 17 pg/mL and from 6.0 to 3.2 μg/dL, respectively. We were able to follow the atypical, slowly progressive course of SAI due to pembrolizumab.

## Introduction

Adrenal insufficiency is a severe disease that could be potentially life-threatening. The common subjective symptoms of adrenal insufficiency include anorexia, nausea/vomiting, and malaise, and clinical and laboratory features include hypotension/shock, hyponatremia, hypoglycemia, hypercalcemia, and hypereosinophilia [1]. The causes of adrenal insufficiency can be classified according to the location into primary adrenal insufficiency, which is caused by disorders of the adrenal gland, and secondary adrenal insufficiency (SAI), which is caused by disorders of the anterior pituitary or hypothalamus [2,3]. The main causes of SAI are pituitary tumors and intracranial diseases such as pituitary apoplexy and hypophysitis [2,3]. In recent years, increasing numbers of patients have developed SAI as an immune-related adverse event (irAE) caused by immune checkpoint inhibitors (ICIs) [4-6]. In most cases, adrenocorticotropic hormone (ACTH) deficiency after administration of ICIs develops acutely, with rapid decreases in ACTH and cortisol levels [6]. However, we experienced a case of slowly progressive SAI that occurred during treatment with pembrolizumab, an anti-programmed cell death-1 (PD-1) antibody for bladder cancer. We were able to observe the onset and relatively slow course of SAI in detail, which progressed monthly, by performing multiple endocrinological evaluations, including loading tests.

## Case presentation

A man underwent resection of a pituitary tumor at the age of 55 years. Preoperative examination revealed no abnormalities in pituitary hormones, and he was diagnosed with a nonfunctioning pituitary neuroendocrine tumor (PiTNET). About a week after transsphenoidal surgery, testosterone was started for postoperative hypogonadism. The basal levels of ACTH and cortisol were 54 pg/mL and 22.3 μg/dL, respectively, and the other pituitary hormones were within normal range after the operation.

At the age of 70, he was diagnosed and underwent transurethral resection for bladder cancer and four cycles of postoperative chemotherapy with gemcitabine and cisplatin. However, 32 weeks later, he was diagnosed with recurrence and lung metastasis, and pembrolizumab was initiated as second-line therapy. When pembrolizumab therapy was initiated, his basal ACTH and cortisol levels were 38 pg/mL and 14.9 μg/dL, respectively, so there could be no apparent adrenal insufficiency, although loading tests were not performed. About three months after initiation of pembrolizumab, which was a few days before the fifth administration, the patient developed anorexia and fatigue. Ten days after receiving the fifth administration, his serum sodium (Na) level had decreased to 120 mEq/L, and he was admitted to the hospital on that day. He had comorbid hypertension and hyperuricemia, for which he was taking olmesartan and febuxostat, respectively. He had a history of alcoholic liver disease but was not a habitual drinker and had no liver damage at the time of admission.

First admission

**Figure 1:**
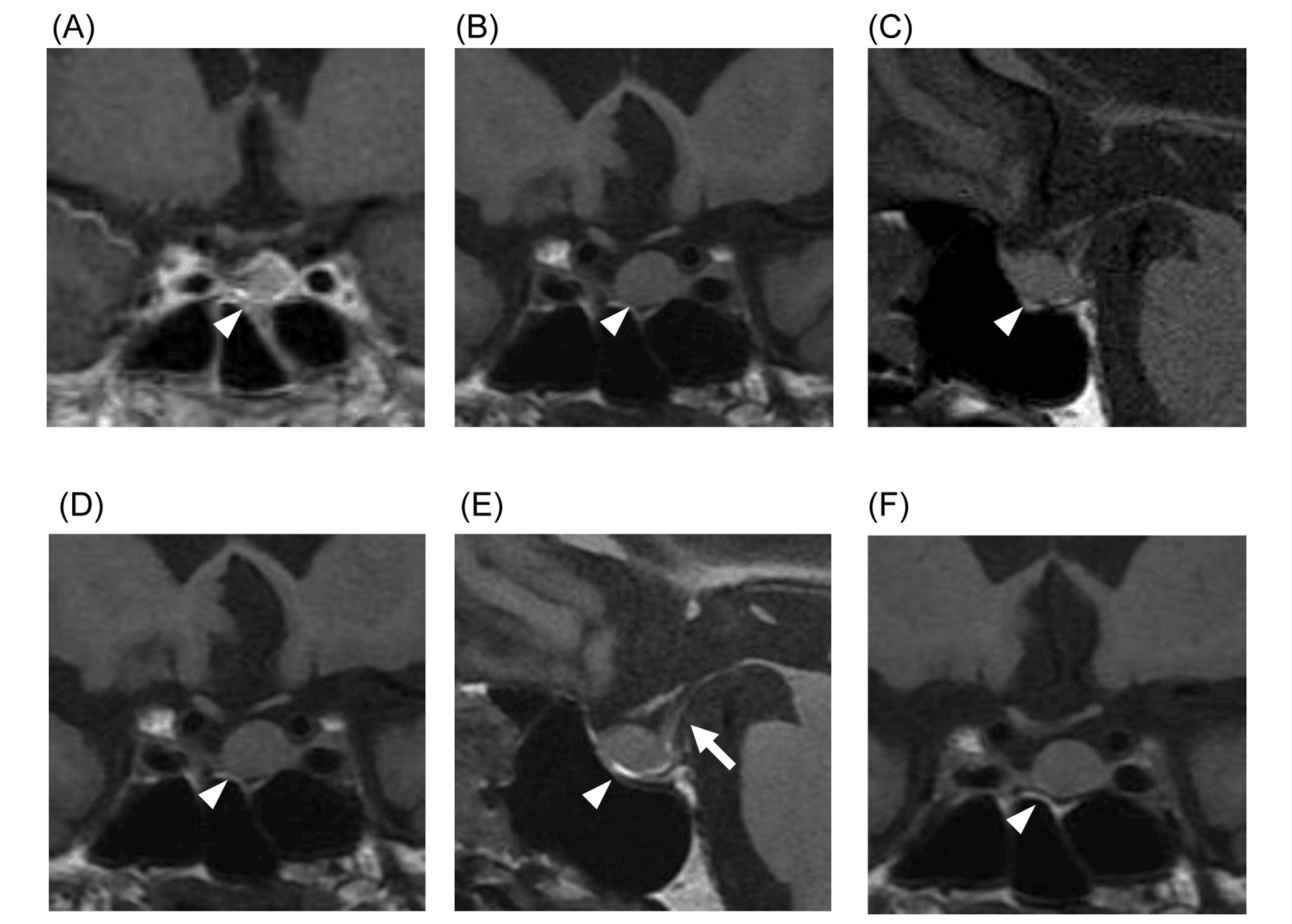
Pituitary T1-weighted MRI. White arrowheads indicate the pituitary gland. (A) Contrast-enhanced image taken 17 years before presentation, three months after surgery. (B) First admission, coronal section. (C) First admission, sagittal section. (D) Second admission, coronal section. (E) Second admission, sagittal section. Triangle marks indicate a pituitary adenoma, and the arrow indicates the pituitary stalk. (F) Contrast-enhanced image taken four months after diagnosis of adrenal insufficiency.

**Figure 2:**
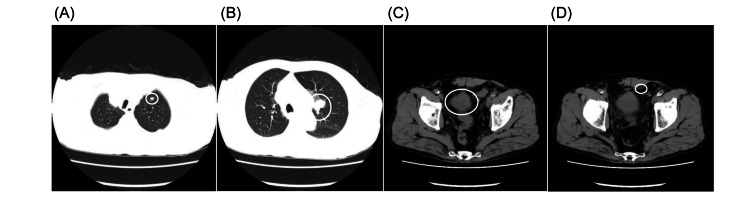
CT scan of the chest and abdomen. Circles indicate (A) lung metastasis, (B) mediastinal lymph node metastasis, (C) primary prostate cancer, and (D) peritoneal dissemination.

On admission, the patient’s consciousness was clear, and vitals were stable (heart rate = 65 beats/min, blood pressure = 132/80 mmHg with/without postural drop, temperature = 35.9℃). No edema was present in the lower extremities, and skin turgor was normal. Table 1 shows the results of blood tests at admission. Blood tests revealed a decreased serum Na level of 120 mEq/L, plasma osmolality of 238 mOsm/kg H2O, and urinary Na of 134 mEq/L, leading to a diagnosis of hypotonic hyponatremia with renal Na excretion. Complete blood count showed bicytopenia (anemia and thrombocytopenia). Testosterone, dehydroepiandrosterone sulfate (DHEA-S), and insulin-like growth factor-1 (IGF-1) revealed low levels, and other endocrine-related hormones were within normal range (Table 1). Magnetic resonance imaging (MRI) of the head showed that the pituitary tumor had changed from 6 × 8 mm after surgery 17 years ago (Figure 1A) to 9 × 11 mm on administration (Figures 1B, 1C). There was no deviation of the pituitary stalk. Thoracoabdominal computed tomography showed slightly increased lung and mediastinal lymph node metastases compared with three months earlier. However, the primary bladder metastasis and peritoneal dissemination had decreased (Figure 2).

**Table 1:** Laboratory data on first admission. WBC, white blood cells; RBC, red blood cells; Hb, hemoglobin; Ht, hematocrit; Plt, platelets; Na, sodium; K, potassium; Cl, chloride; AST, aspartate aminotransferase; ALT, alanine aminotransferase; γGTP, γ-glutamyl transpeptidase; LDH, lactate dehydrogenase; ALP, alkaline phosphatase; UA, uric acid; UN, urea nitrogen; Cr, creatinine; TP, total protein; Alb, albumin; HDL-Chol, high-density lipoprotein cholesterol; LDL-Chol, low-density lipoprotein cholesterol; TG, triglyceride; TSH, thyroid-stimulating hormone; FT4, free thyroxine; ACTH, adrenocorticotropic hormone; GH, growth hormone; IGF-1, insulin-like growth factor-1; LH, luteinizing hormone; FSH, follicle-stimulating hormone; PRL, prolactin; AVP, vasopressin; DHEA-S, dehydroepiandrosterone sulfate.

Item	Patient	Reference
WBC	5550/μL	3300 – 9400/μL
Neutrophil	58.2 %	40.0 – 73.0%
Lymphocyte	27.9 %	18.0 – 52.0%
Monocyte	7.6 %	2.2 – 10.0%
Eosinophil	5.2 %	0.0 – 7.0%
Basophil	1.1 %	0.0 – 2.0%
RBC	367×10^4^/μL	440 – 560 ×104/μL
Hb	12.4 g/dL	13.8 – 17.0 g/dL
Ht	33.7%	41.0 – 51.0%
Plt	11.0×10^4^/μL	13.0 – 32.0 ×104/μL
Na	120 mEq/L	138 – 145 mEq/L
K	4.1 mEq/L	3.6 – 4.8 mEq/L
Cl	100 mEq/L	100 – 108 mEq/L
AST	30 U/L	40 U/L
ALT	21 U/L	40 U/L
γGTP	27 U/L	12 – 69 U/L
LDH	141 U/L	124 – 222 U/L
ALP	67 U/L	38 – 113 U/L
UA	5.5 U/L	3.6 – 7.2 mg/dL
UN	16 mg/dL	7 – 22 mg/dL
Cr	0.75 mg/dL	0.6 – 1.2 mg/dL
TP	6.8 g/dL	6.4 – 8.1 g/dL
Alb	4.1 g/dL	3.6 – 4.7 g/dL
HDL-Chol	36 mg/dL	40 – 80 mg/dL
LDL-Chol	87 mg/dL	140 mg/dL
TG	169 mg/dL	150 mg/dL
Glucose	105 mg/dL	70 – 110 mg/dL
Osmotic pressure	238 mOsm/kgH2O	280 – 290 mOsm/kgH2O
TSH	3.78 μU/mL	0.61 – 4.23 μU/mL
FT4	1.2 ng/dL	0.8 – 1.7 ng/dL
ACTH	35 pg/mL	7 – 63 pg/mL
Cortisol	7.6 μg/dL	4.0 – 18.3 μg/dL
GH	0.23 ng/mL	0.0 – 2.47 ng/mL
IGF-1	32 ng/mL	58 – 198 ng/mL
LH	2.9 mU/mL	1.7 – 10.0 mU/mL
FSH	12.8 mU/mL	1.5 – 17.2 mU/mL
Testosterone	1.46 ng/mL	1.87 – 9.02 ng/mL
PRL	6.7 ng/mL	3.7 – 30.1 ng/mL
AVP	0.90 pg/mL	2.8 pg/mL
Renin activity	2.4 ng/mL/h	0.2 – 2.7 ng/mL/h
Aldosterone	36.2 pg/mL	10.4 – 142.3 pg/mL
DHEA-S	16.4 pg/mL	34.5 – 568.9 g/dL

**Figure 3:**
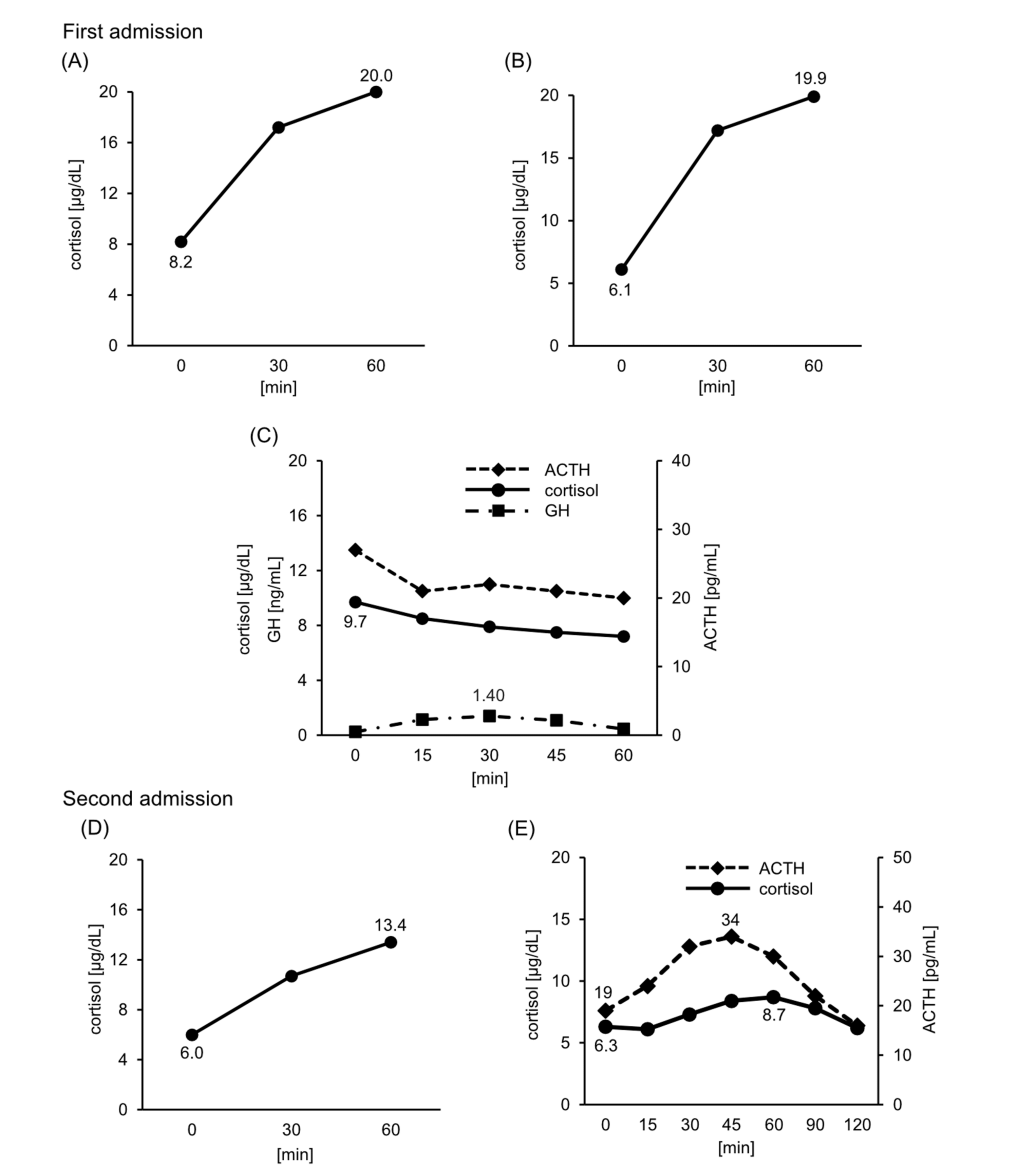
The results of loading tests. (A) 250-μg ACTH test performed on day six of hospitalization. (B) 1-μg ACTH challenge test performed on day 12 of hospitalization. (C) GHRP-2 loading test performed on day 13 of hospitalization. (D) Rapid ACTH loading test performed on day 34 of hospitalization. (E) CRH test performed on day 35 of hospitalization. ACTH, adrenocorticotropic hormone; GH, growth hormone; GHRP-2, growth hormone-releasing peptide-2; CRH, corticotropin-releasing hormone.

The serum Na level gradually improved with the administration of oral hydrocortisone at 15 mg/day and sodium chloride tablets at 3 g/day. A rapid 250-μg ACTH loading test was performed on day five of hospitalization, 24 hours after withdrawal of hydrocortisone, and the peak cortisol level was 20 μg/dL (normal response) (Figure 3A). A 1-μg ACTH test showed a cortisol peak of 19.9 µg/dL (normal response) (Figure 3B). The additional test was not performed because we determined that the patient did not have adrenal insufficiency based on the ACTH loading tests [7], and the hydrocortisone administration was discontinued. Because the serum vasopressin on admission was above the sensitivity level (Table 1), syndrome of inappropriate antidiuretic hormone secretion or salt-loss nephropathy was suspected, and salt loading and restricted drinking water at 15 mL/kg/day were continued. A growth hormone-releasing peptide-2 (GHRP-2) loading test was performed on day 12 of hospitalization because of the low level of IGF-1, and the result showed a low response with a peak growth hormone (GH) level of 1.4 ng/mL (Figure 3C). Together with the presence of hypogonadotropic hypogonadism and a pituitary tumor, these findings led to the diagnosis of adult GH deficiency. ACTH and cortisol were also measured simultaneously with the GHRP-2 loading test, but they were unresponsive (Figure 3C). The serum sodium level did not decrease after discontinuing hydrocortisone, so syndrome of inappropriate antidiuretic hormone secretion (SIADH) was most suspected. The patient was discharged with salt loading.

Second admission

A total of 14 days after discharge, the patient was readmitted to the hospital for loss of appetite, worsening fatigue, and hyponatremia. His consciousness was clear, and his blood pressure, pulse, and temperature were within the normal range. Like in the first admission, his plasma osmolality and urinary Na level indicated hypotonic hyponatremia with renal Na excretion. Unlike the initial admission, the eosinophil count had increased to 713/μL. Endocrine parameters showed a decrease in cortisol to 2.2 μg/dL and a mild increase in thyroid-stimulating hormone (TSH) to 4.84 μIU/mL, with other tests showing no significant difference compared to the first (Table 2). MRI of the head showed that the tumor diameter was 9 × 11 mm, and findings were similar to the first admission (Figures 3D, 3E). Because of suspected new-onset SAI, hydrocortisone supplementation was initiated. A rapid ACTH loading test was performed again four weeks after the first test, and the patient was hyporesponsive with a basal cortisol level of 6.0 μg/dL and peak level of 13.4 μg/dL (Figure 3D). In addition, a corticotropin-releasing hormone (CRH) test showed a low response with a basal cortisol level of 6.3 μg/dL and a peak value of 8.7 μg/dL, as well as a basal ACTH level of 19 pg/mL and a peak value of 34 pg/mL (Figure 3E). From these findings, the patient was diagnosed with SAI.

**Table 2:** Laboratory data on second admission. WBC, white blood cells; RBC, red blood cells; Hb, hemoglobin; Ht, hematocrit; Plt, platelets; Na, sodium; K, potassium; Cl, chloride; AST, aspartate aminotransferase; ALT, alanine aminotransferase; γGTP, γ-glutamyl transpeptidase; LDH, lactate dehydrogenase; ALP, alkaline phosphatase; UA, uric acid; UN, urea nitrogen; Cr, creatinine; TP, total protein; Alb, albumin; HDL-Chol, high-density lipoprotein cholesterol; LDL-Chol, low-density lipoprotein cholesterol; TG, triglyceride; TSH, thyroid-stimulating hormone; FT4, free thyroxine; ACTH, adrenocorticotropic hormone; GH, growth hormone; IGF-1, insulin-like growth factor-1; LH, luteinizing hormone; FSH, follicle-stimulating hormone; PRL, prolactin; AVP, vasopressin; DHEA-S, dehydroepiandrosterone sulfate.

Item	Patient	Reference
WBC	8590/μL	3300 – 9400/μL
Neutrophil	58.3%	40.0 – 73.0%
Lymphocyte	24.1%	18.0 – 52.0%
Monocyte	8.6%	2.2 – 10.0%
Eosinophil	8.3%	0.0 – 7.0%
Basophil	0.7%	0.0 – 2.0%
RBC	348×104/μL	440 – 560 ×104/μL
Hb	11.7 g/dL	13.8 – 17.0 g/dL
Ht	32.7%	41.0 – 51.0%
Plt	15.3×104/μL	13.0 – 32.0 ×104/μL
Na	122 mEq/L	138 – 145 mEq/L
K	4.3 mEq/L	3.6 – 4.8 mEq/L
Cl	102 mEq/L	100 – 108 mEq/L
AST	21 U/L	40 U/L
ALT	12 U/L	40 U/L
γGTP	29 U/L	12 – 69 U/L
LDH	143 U/L	124 – 222 U/L
ALP	65 U/L	38 – 113 U/L
UA	5.5 mg/dL	3.6 – 7.2 mg/dL
UN	12 mg/dL	7 – 22 mg/dL
Cr	0.79 mg/dL	0.6 – 1.2 mg/dL
TP	6.8 g/dL	6.4 – 8.1 g/dL
Alb	4.3 g/dL	3.6 – 4.7 g/dL
HDL-Chol	34 mg/dL	40 – 80 mg/dL
LDL-Chol	116 mg/dL	140 mg/dL
TG	150 mg/dL	150 mg/dL
Glucose	96 mg/dL	70 – 110 mg/dL
Osmotic pressure	246 mOsm/kgH2O	280 – 290 mOsm/kgH2O
TSH	4.84 μU/mL	0.61 – 4.23 μU/mL
FT4	1.1 ng/dL	0.8 – 1.7 ng/dL
ACTH	18 pg/mL	7 – 63 pg/mL
Cortisol	2.2 μg/dL	4.0 – 18.3 μg/dL
GH	0.30 ng/m	0.0 – 2.47 ng/mL
IGF-1	33 ng/mL	58 – 198 ng/mL
LH	3.4 mU/mL	1.7 – 10.0 mU/mL
FSH	12.7 mU/mL	1.5 – 17.2 mU/mL
Testosterone	1.05 ng/mL	1.87 – 9.02 ng/mL
PRL	6.5 ng/mL	3.7 – 30.1 ng/mL
AVP	1.0 pg/mL	2.8 pg/mL
Renin activity	2.6 ng/mL/h	0.2 – 2.7 ng/mL/h
Aldosterone	20.6 pg/mL	10.4 – 142.3 pg/mL
DHEA-S	12.2 μg/dL	34.5 – 568.9 μg/dL

Outcome and follow-up

**Figure 4:**
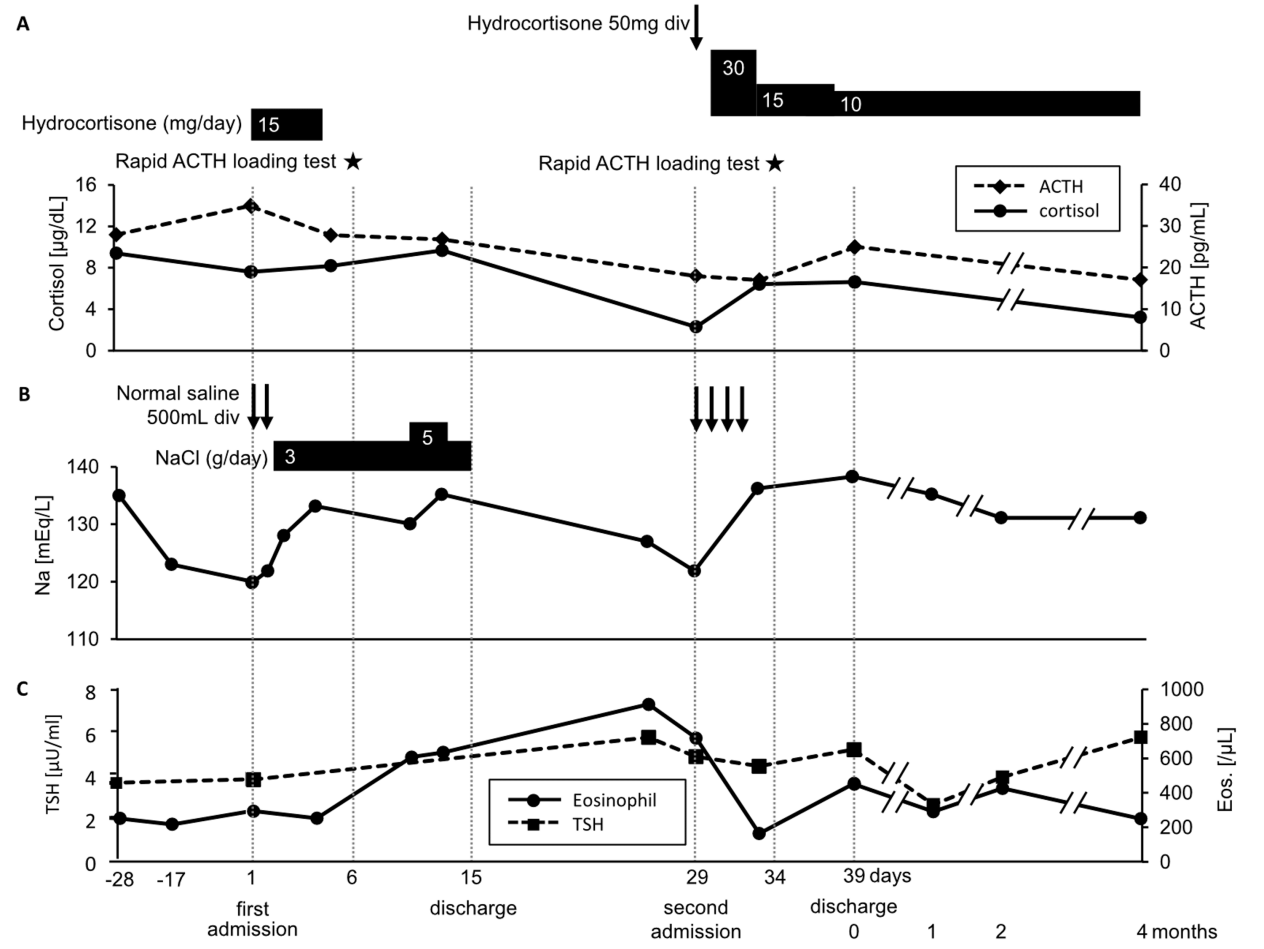
Clinical course. (A) Trends of early morning ACTH and cortisol levels and administration of hydrocortisone. Stars indicate when the rapid ACTH loading tests were performed. (B) Trends of serum sodium and administration of NaCl. (C) Trends of eosinophil count and TSH level. Arrows indicate intravenous administration, and bars indicate oral administration. The horizontal axis indicates the number of days, with day one as the date of initial admission. After the second discharge, the number represents the number of months since discharge. ACTH, adrenocorticotropic hormone; NaCl, sodium chloride; TSH, thyroid-stimulating hormone.

Because the patient had residual adrenocortical function, the hydrocortisone was reduced to 10 mg/day, and the patient was discharged after the serum Na level was maintained at approximately 135 mEq/L. Four months after diagnosis, the patient’s serum cortisol and ACTH levels further decreased (Table 3). Hydrocortisone was continued at 10 mg/day, the serum Na level remained stable, and head MRI revealed no changes (Figure 1F). The clinical course from initial admission to readmission and after discharge is shown in Figure 4. All blood tests were performed in the morning after approximately 24 hours of hydrocortisone withdrawal. His adrenocortical function did not recover, and he died of bladder cancer about a year after diagnosis.

**Table 3:** Time course of plasma ACTH, serum cortisol, and DHEA-S. ACTH, adrenocorticotropic hormone; DHEA-S, dehydroepiandrosterone sulfate.

Item	First admission	Second admission	4 months after the second discharge
ACTH (pg/mL)	28	19	17
Cortisol (μg/dL)	8.2	6.0	3.2
DHEA-S (μg/dL)	16.4	12.2	18.4

## Discussion

This report describes SAI during treatment with pembrolizumab after surgery for bladder cancer in a case with a past history of a nonfunctioning pituitary tumor. SAI was diagnosed after repeated hospitalizations for hyponatremia. This case report is novel in that the course of SAI progressed relatively slowly on a monthly basis after onset but did not develop into a complete cortisol deficiency. We were able to evaluate this clinical course with multiple measurements of adrenal function-related parameters and loading tests.

Onset of secondary adrenal insufficiency

In this case, the patient responded well to the first rapid ACTH loading test but poorly to the second, suggesting that the disease had progressed in the intervening weeks. Moreover, even before rehospitalization, the patient presented with low serum Na level, eosinophilia, and mildly elevated TSH, which were suggestive of adrenal insufficiency [8]. Therefore, we suspected that the adrenal insufficiency may have developed prior to rehospitalization. This case report highlights the blood test was performed for eosinophil count and TSH weekly, which enabled early diagnosis before the onset of these symptoms. Additionally, in cases of ACTH deficiency caused by ICIs, ACTH and cortisol levels are often markedly low at the time of diagnosis because of sudden-onset SAI [9]. In a case report in which ACTH and cortisol were followed up with blood tests twice a month after pembrolizumab administration [10], the patient acutely developed SAI within approximately two weeks, and the cortisol level had already decreased to 1.4 μg/dL at diagnosis. The authors also reviewed 32 patients who developed SAI after receiving a PD-1 antibody product, and in all patients, the cortisol level was <4 μg/dL at diagnosis [10]. By contrast, a case report of SAI due to pituitary gland damage after pembrolizumab administration discussed the possibility of SAI progressing on a monthly basis [11,12]. Our patient had an early morning basal cortisol level of 6.0 μg/dL at diagnosis, which decreased to 3.2 μg/dL four months later. This process of cortisol decline over several months is similar to that in the above-mentioned previous reports.

Evaluation of the hypothalamic-pituitary-adrenal (HPA) axis by the GHRP-2 test

The GHRP-2 loading test can also reportedly elicit a response in the HPA axis [13-15]. ACTH and cortisol levels are elevated in healthy individuals, whereas the cortisol response is decreased in patients with SAI. In addition, the ACTH response is preserved in patients with hypothalamic disease but decreased in those with pituitary disease, suggesting that GHRP-2 may stimulate the pituitary gland and promote ACTH secretion [13]. In our patient, both ACTH and cortisol were unresponsive in the GHRP-2 loading test, supporting SAI caused by pituitary insufficiency. Endocrinological data show that in men with nonfunctioning pituitary tumors, the cortisol peak values in the GHRP-2 loading test correlate well with the insulin tolerance test [16]. In this report, the peak cortisol level of 15.8 µg/dL in the GHRP-2 loading test was the most accurate predictor of a normal cortisol response in the insulin tolerance test. Therefore, the low peak cortisol level of 9.7 mg/dL in the GHRP-2 loading test on day 12 might have provided an opportunity to suspect the development of SAI earlier.

Cause of secondary adrenal insufficiency

Potential causes of SAI in this case were an irAE due to the anti-PD-1 antibody pembrolizumab and a mass effect due to the pituitary tumor. Pembrolizumab-induced pituitary disorder has an average onset time of 18 to 44 weeks after the start of pembrolizumab administration [4]. Our case is consistent with this, as the onset was estimated to have occurred at 18 to 21 weeks. As another possibility, SAI caused by a tumor-induced mass effect may develop as the tumor grows [8,17,18]. In one study, the prevalence of SAI increased with tumor size: 38%, 47%, and 71% with diameters of ≦19 mm, 20-29 mm, and ≧30 mm, respectively [8]. In another study, the pituitary tumors that occupy the sella space and compress the walls on both sides of the cavernous sinus with Knosp grades 1-3 on both sides had a higher risk of developing SAI [17]. In this case, the tumor grew very slowly from 6 × 8 mm to 9 × 11 mm during the 17-year period after the tumor resection, and it did not grow during the progression of SAI. Knosp grades were 0 on both sides, with no changes after pituitary tumor resection. In pituitary tumors, anterior pituitary hormone production is impaired in the order of growth hormone, TSH, and ACTH [19]. In the present case, the basal TSH level was above the reference range, which does not support SAI due to a pituitary tumor. Therefore, we considered the possibility of SAI due to a pituitary tumor to be unlikely. Taken together, these considerations lead us to suggest that irAE was most likely.

## Conclusions

We experienced a case of SAI diagnosed after the onset of hyponatremia during treatment with pembrolizumab, which progressed over a month-long clinical course. The residual cortisol secretion at the time of diagnosis and the progression over a month-long period of SAI were unique to this case compared with previously reported cases of SAI due to irAEs. Because there are rare cases of slow progression of SAI due to irAEs, it is important to repeat the loading test when the onset of SAI is suspected.

## References

[1] Puar TH, Stikkelbroeck NM, Smans LC, Zelissen PM, Hermus AR (2016). Adrenal crisis: still a deadly event in the 21st century. Am J Med.

[2] Husebye ES, Pearce SH, Krone NP, Kämpe O (2021). Adrenal insufficiency. Lancet.

[3] Bancos I, Hahner S, Tomlinson J, Arlt W (2015). Diagnosis and management of adrenal insufficiency. Lancet Diabetes Endocrinol.

[4] Stelmachowska-Banaś M, Czajka-Oraniec I (2020). Management of endocrine immune-related adverse events of immune checkpoint inhibitors: an updated review. Endocr Connect.

[5] Lu J, Li L, Lan Y, Liang Y, Meng H (2019). Immune checkpoint inhibitor-associated pituitary-adrenal dysfunction: a systematic review and meta-analysis. Cancer Med.

[6] Wang PF, Chen Y, Song SY (2017). Immune-related adverse events associated with anti-PD-1/PD-L1 treatment for malignancies: a meta-analysis. Front Pharmacol.

[7] Kumar R, Wassif WS (2022). Adrenal insufficiency. J Clin Pathol.

[8] Mukai K, Kitamura T, Tamada D, Murata M, Otsuki M, Shimomura I (2016). Relationship of each anterior pituitary hormone deficiency to the size of non-functioning pituitary adenoma in the hospitalized patients. Endocr J.

[9] Nguyen H, Shah K, Waguespack SG (2021). Immune checkpoint inhibitor related hypophysitis: diagnostic criteria and recovery patterns. Endocr Relat Cancer.

[10] Hinata Y, Ohara N, Sakurai Y (2021). Isolated adrenocorticotropic hormone deficiency associated with severe hyperkalemia during pembrolizumab therapy in a patient with ureteral cancer and an ileal conduit: a case report and literature review. Am J Case Rep.

[11] Yamagata S, Kageyama K, Takayasu S, Asari Y, Makita K, Terui K, Daimon M (2019). Progression of hypopituitarism and hypothyroidism after treatment with pembrolizumab in a patient with adrenal metastasis from non-small-cell lung cancer. Intern Med.

[12] Matsushiro M, Shibue K, Osawa K, Hamasaki A (2024). Isolated adrenocorticotropic hormone (ACTH) deficiency as an immune-related adverse event following combination immune checkpoint inhibitor therapy. Cureus.

[13] Arimura H, Hashiguchi H, Yamamoto K (2016). Investigation of the clinical significance of the growth hormone-releasing peptide-2 test for the diagnosis of secondary adrenal failure. Endocr J.

[14] Kimura T, Shimatsu A, Arimura H (2010). Concordant and discordant adrenocorticotropin (ACTH) responses induced by growth hormone-releasing peptide-2 (GHRP-2), corticotropin-releasing hormone (CRH) and insulin-induced hypoglycemia in patients with hypothalamopituitary disorders: evidence for direct ACTH releasing activity of GHRP-2. Endocr J.

[15] Kano T, Sugihara H, Sudo M (2010). Comparison of pituitary-adrenal responsiveness between insulin tolerance test and growth hormone-releasing peptide-2 test: a pilot study. Peptides.

[16] Hayakawa T, Kitamura T, Tamada D (2018). Evaluation of hypothalamic-pituitary-adrenal axis by the GHRP2 test: comparison with the insulin tolerance test. J Endocr Soc.

[17] Oshino S, Saitoh Y, Kinoshita M, Mukai K, Otsuki M, Kishima H (2021). Characteristics of nonfunctioning pituitary adenomas that cause secondary adrenal insufficiency. World Neurosurg.

[18] Almistehi WM, Vaninetti N, Mustafa S (2020). Secondary pituitary hormonal dysfunction patterns: tumor size and subtype matter. Pituitary.

[19] Caturegli P, Newschaffer C, Olivi A, Pomper MG, Burger PC, Rose NR (2005). Autoimmune hypophysitis. Endocr Rev.

